# Over 25 years of decrypting PIN-mediated plant development

**DOI:** 10.1038/s41467-024-54240-y

**Published:** 2024-11-15

**Authors:** Christian Luschnig, Jiří Friml

**Affiliations:** 1 Department of Applied Genetics and Cell Biology, Institute of Molecular Plant Biology, BOKU University, Wien, Austria; 2 Institute of Science and Technology Austria (ISTA), Klosterneuburg, Austria

**Keywords:** Plant sciences, Cell biology

## Abstract

Identification of PIN exporters for auxin, the major coordinative signal in plants, some 25 years ago, signifies a landmark in our understanding of plant-specific mechanisms underlying development and adaptation. Auxin is directionally transported throughout the plant body; a unique feature already envisioned by Darwin and solidified by PINs’ discovery and characterization. The PIN-based auxin distribution network with its complex regulations of PIN expression, localization and activity turned out to underlie a remarkable multitude of developmental processes and represents means to integrate endogenous and environmental signals. Given the recent anniversary, we here summarize past and current developments in this exciting field.

## Abstract (web summary)

In this perspective, the authors summarize some 25 years of research on PIN auxin transport proteins and reflect on consequences for the understanding of cellular processes underlying plant development and adaptation.

## Introduction

Sometime in 1996 at the Max Planck Institute for Plant Breeding in Cologne, Germany, Leo Gälweiler, an enthusiastic PhD student intended to document some not too exciting experimental outcomes. While queuing for the institute’s photographer, he chatted with Ellen Wisman, a colleague waiting to photograph a strange looking Arabidopsis plant. This was the *pin-formed1* (*pin1*) mutant with its typical knitting needle-like stems and, despite similar mutants having already been described and linked to the transport of the phytohormone auxin^1^, the gene underlying this fascinating phenotype remained unknown. After realizing that Ellen’s scientific interests lie elsewhere, Leo, intrigued by the mutant’s persistent mystery, saw a chance to upgrade his PhD project. A deal was struck. Neither Leo nor Ellen realized at that time that their chance encounter would be a turning point in a long quest to understand auxin and a multitude of its roles in physiology and development. They also did not know that at the same time at different places, other scientists made similar fateful decisions with their respective Arabidopsis mutants; At the Whitehead Institute/MIT (Cambridge, USA), bulking up neglected seed stocks of transposon-tagging lines led to the identification of a root agravitropic *ethylene insensitive root 1* (*eir1*) allele, whilst positional cloning approaches were initiated at the University of Wisconsin (USA) to go after *agravitropic* (*agr*). Finally, at the Nara Institute of Science and Technology (Japan), colleagues became interested in mutants defective in wavy root growth, such as *wavy roots 6* (*wav6*). Independent efforts by all these groups led to the identification of an unknown, plant-specific family of proteins with limited similarity to membrane transporters. Five papers published in 1998 reported these exciting findings^2–6^. The saga of the PIN auxin transporters has just begun.

The saga of auxin itself dates back much further—to Charles Darwin and his son Francis. While most biologists share an admiration for Darwin’s theory of evolution, researchers working in the field of phytohormones are blessed additionally, as Darwin devoted an entire book to this phenomenon, meticulously describing the movement of plant organs and hypothesizing on a mobile signal defining such responses. Whilst rather a non-seller, ‘The Power of Movements in Plants’^7^ became an inspiration, when research conducted some 50 years later, came up with the auxins. Auxins—including their main representative, the simple organic compound indole-3-acetic acid (IAA)—were found to move along plant body axes and were sufficient to promote organ bending, thus fulfilling criteria of the postulated growth-promoting signal^8^. It became also clear early on that auxin’s function is intimately linked to its directional transport. Especially, discovery of chemical inhibitors of this polar auxin transport (PAT) and their high affinity binding to plasma membrane (PM) fractions led to the idea that PAT might require transport sites located at the PM^9^.

**Fig. 1:**
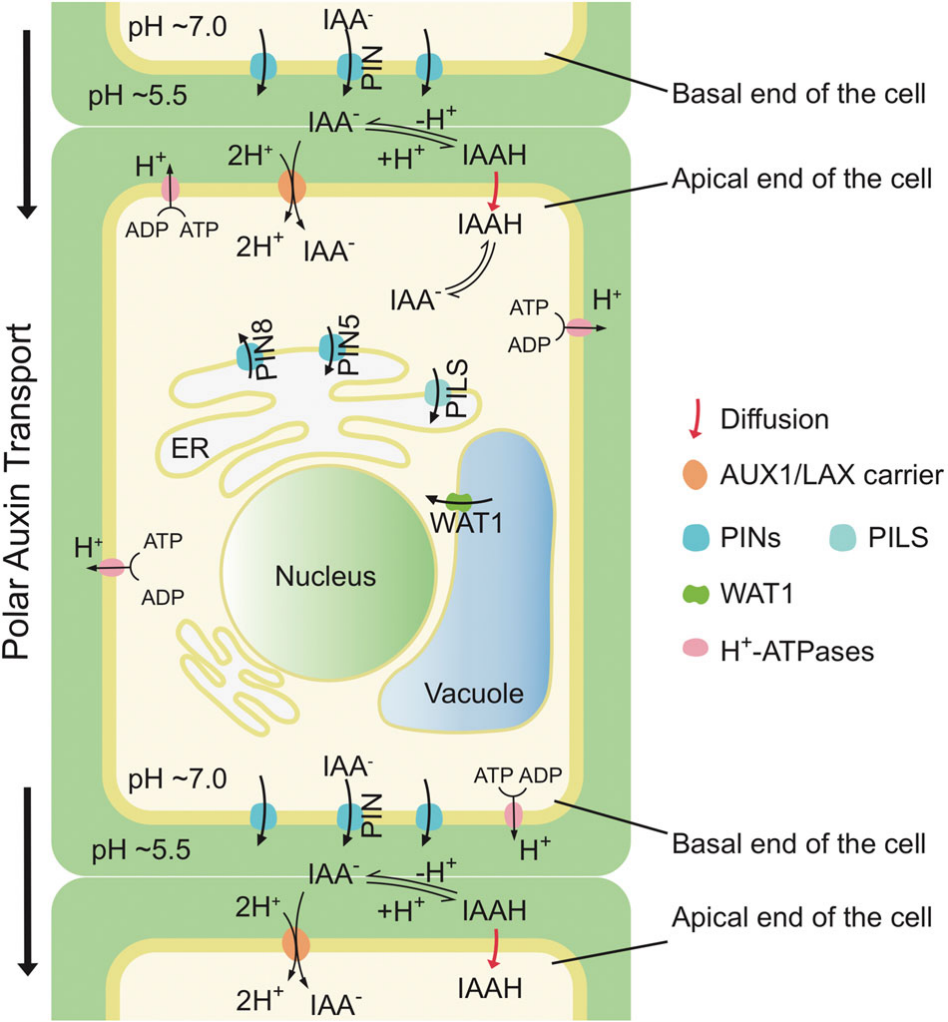
PAT as postulated by the chemiosmotic hypothesis, is perfectly matched by PIN functions in the intercellular transport of auxin. Cellular uptake of protonated IAAH from a moderately acidic apoplast, which is maintained by PM-ATPase activity, might proceed via passive diffusion across the plasma membrane (red arrow) or via AUX1 auxin/2H^+^ symport activity. The neutral cytoplasmic pH results in IAAH deprotonation, preventing further diffusion of the growth regulator. PIN positioning at a polar PM domain, as exemplified by the basal domain in this image, will define the sites of IAA^−^ efflux into the apoplast, which is followed by auxin reuptake into the adjacent cell. Ultimately, the directionality of intercellular auxin transport from top to bottom in the cell file on display (black arrows) is defined by a strictly basal PIN polarity in a sequence of adjacent cells. Next to PM-localized canonical PIN proteins, ER-localized noncanonical PINs as well as PILS proteins are believed to impact on intracellular auxin homeostasis by shaping compartmentalization of auxin and/or auxin conjugates. The directionality of such transport at the ER, particularly in case of PIN8 remains to be determined. Additional intracellular auxin transport activities have been characterized for tonoplast-localized WALLS ARE THIN 1 (WAT1).

It was the work by Rubery, Sheldrake, and Raven^10,11^ that put these observations into perspective, by formulating the ‘chemiosmotic hypothesis’ for PAT, outlining the key mechanistic features of this process (Box 1 and Fig. 1). These remarkable insights however remained theoretical for decades until the emergence of *Arabidopsis thaliana* as the genetic model in the 1990s, when joint efforts of numerous labs led to first molecular insights into many processes including auxin transport. For example, mutants in *AUXIN RESISTANT 1* (*AUX1*) exhibit an auxin-resistant agravitropic root growth, and the corresponding gene product turned out to function as an auxin uptake carrier^12,13^. Other agravitropic mutants such as *agr*^2,6^, *eir1*^4^ or *wav6*^14^ appeared also very promising, as PAT inhibition always leads to agravitropic growth. However, it was the characteristic, above-ground *pin1* mutant phenotype, with its knitting pin-like structures in place of differentiated inflorescences, which held the most promise^1^. These phenotypes can be perfectly phenocopied, when growing wild type *Arabidopsis* in the presence of certain auxin transport inhibitors, such as 1-*N*-Naphthylphthalamic acid (NPA)^1^. Consistently, *pin1* mutants show auxin transport deficiencies as inferred from the distribution of ^14^C-labelled IAA^1^. Thus, identification of the genes behind the *pin1* or the root agravitropic mutants were expected to reveal components of the auxin transport machinery.

A combination of tedious cloning approaches and serendipity led to an almost simultaneous identification of the first *PIN* loci. Molecular cloning of *agr*, *eir1*, and *wav6* mutants and initial functional characterization of the corresponding gene products ultimately revealed that *AGR*, *EIR1*, *PIN2,* and *WAV6* loci all represent the very same locus. Further, the original description of the *PIN1* gene made it clear that AGR/EIR1/PIN2/WAV6 and PIN1 belong to the same protein family with some limited resemblance to members of the bile/arsenite/riboflavin membrane transporter family, hinting at transport functions for PINs^2–6^.

Box 1 Chemical and cellular requirements for directional intercellular auxin transport Chemical and cellular requirements for directional intercellular auxin transport, as postulated in the chemiosmotic hypothesis for PAT

i. Polar auxin transport occurs in a cell-to-cell fashion with auxin released from one cell taken up by its adjacent cell(s)
ii. intracellular IAA accumulation depends on a pH and/or electrical gradient that is maintained across the PM
iii. cellular uptake of IAA is facilitated by the rather acidic pH of the apoplast, leaving a considerable fraction of extracellular IAA in its protonated form, suitable for passage through the non-polar membrane bilayer
iv. the higher intracellular pH causes IAA deprotonation, limiting further diffusion through membrane barriers, thereby trapping the IAA^−^ anion in the cell’s interior
v. further transport of auxin into any neighbouring cells therefore requires the presence of a specific efflux carrier
vi. asymmetry in efflux carrier localization restricted to defined polar PM domains would define the directionality of further transport

### To-be-or-not-to-be auxin exporters

Initial work on PIN1 and PIN2 supported a function as auxin exporters. The topology with ten transmembrane domains was consistent with a transporter or channel function. Basal PM domain localization of PIN1 in the stem^3^ and opposite apical localization of PIN2 in the root epidermis^5^ matched known rootward and shootward auxin transport routes, compromised in the corresponding mutants or upon chemical inhibition of PAT^1,15^. Furthermore, PIN2, when expressed in yeast led to a reduced accumulation of auxinic compounds^2^, and conveyed more resistance to a toxic structural auxin analogue^4^. With identification of additional PIN proteins (PIN3, PIN4, and PIN7), it turned out that their subcellular localization and corresponding mutant phenotypes are consistent with PINs’ function as auxin exporters. As a further consequence, roles for PIN-mediated auxin transport in highly diverse processes, such as gametophyte and embryo development^16,17^, lateral organ formation and phyllotaxis^18^, leaf venation^19^, or control of seed dispersal^20^ have been firmly established. Additional support came from the prominent deviations in auxin distribution in *pin* mutants, as inferred from the activities of auxin-responsive reporters^4,21–24^. Despite all these observations being consistent with PINs acting as auxin exporters, they received strong contestants for this job – the ATP-driven ABCB1 and ABCB19 (ATP-BINDING CASSETTE-B) transporters^25^. Although the corresponding *abcb* mutants showed phenotypes differing from those of *pin* mutants or caused by inhibition of PAT, they were shown to be targeted by PAT inhibitors and to export auxin, when expressed heterologously^26^.

A few years later, accumulation assays using radioactively labelled auxins, inferred PIN auxin export capabilities when expressed in tobacco cultured cells, or yeast as well as mammalian cells^27^. Additional confirmation of PIN transport function came later from *Xenopus* oocyte experiments, which also revealed the essential need for PIN phosphorylation by AGCVIII-type kinases. Only in presence of the kinase, selected PINs would mediate auxin export from oocytes^28^. Such PIN phosphorylation is crucial for PIN activation in plants as well, as exemplified by AGVIII-type kinase PROTEIN KINASE ASSOCIATED WITH BRX (PAX), which activates PIN1 in root protophloem cells. PAX at the PM in turn is inhibited by its interaction with BREVIS RADIX (BRX)^29^.

Entirely unexpected was a finding that some of the Arabidopsis PINs, while still functioning as auxin transporters, are localized at the membrane of the Endoplasmic Reticulum (ER), namely PIN5 and PIN8^30,31^. ER-localized PINs are commonly characterized by a much shorter central hydrophilic loop than found in canonical, PM-localized PINs. The role of the ER-localized PINs as well as of PIN6 that can be found both, at the ER and the PM^32,33^ remains vague, but presumably relates to intracellular auxin metabolism and homeostasis. Whatever their function may be, transport of auxin or auxin metabolites across the ER membrane seems essential, since besides ER-localized PINs also PIN-LIKES (PILS) transporters for auxinic compounds localize there^34^.

### A long way to structure and mechanism of auxin translocation

Genetic, physiological, and cell biological experiments provided strong cumulative evidence for PINs acting as auxin exporters. However, as ever so often, the lack of insights into protein structure hampered the mechanistic understanding of PIN-mediated auxin translocation. Canonical PINs with their large central hydrophilic loop represented a particular challenge to structural studies due to their poorly conserved and disordered loop domain. In silico structural predictions and homology-based modelling for canonical PINs supported originally formulated models, in which two conserved domains of five transmembrane (TM) helices each, are separated by a less conserved hydrophilic loop domain of variable length, but provided only limited insights into the structure and topology^35,36^. Nevertheless, protease mapping and detergent-sensitive immunostaining consistently predicted the central loop facing the cytoplasm, and the N- and C-termini likely to extend into the apoplast^35^. In accordance, a majority of known regulatory sites and peptide motifs locate to this central loop domain.

Native protein separation of membrane protein fractions and in vivo crosslinking identified homo-oligomers made up of PIN1 and other canonical PINs^37,38^, overall implying formation of PIN oligomers under physiological conditions. Notably, NPA and quercetin, a naturally occurring PAT regulator, rendered PIN dimers more stable, suggesting that these inhibitory compounds force PINs into a conformation unfavourable for auxin translocation across the PM^37^. The NPA effects on PIN configuration and activity either *in planta* or in heterologous systems such as oocytes, suggested a direct association of NPA with PINs, dispelling with a decade-old notion of an NPA-binding protein as a distinct element of the auxin transport machinery^37,38^. All these spectacular insights into PIN function and configuration represented a prelude for the things that were to come—the deciphering of the PIN structure, almost quarter a century after their initial discovery.

**Fig. 2:**
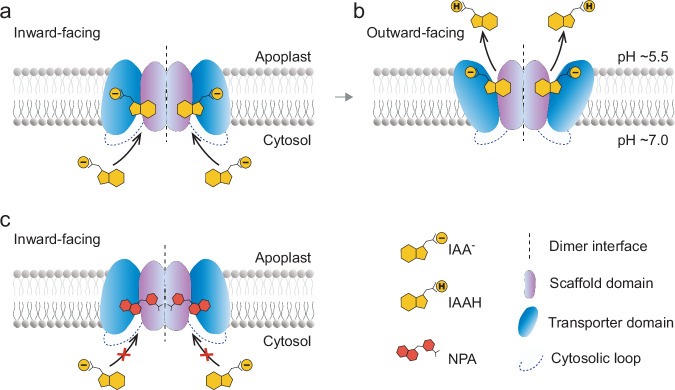
Structure and configuration of membrane-intrinsic PINs. **a** Canonical and non-canonical PINs, both have been identified as symmetrically arranged homodimers, with the scaffold domains (purple) of both monomers facing each other at the dimer interface. In an inward-open configuration, the transporter domain (blue) of each of the monomers adopts a configuration, which allows for entry of an IAA^−^ anion (yellow) into the substrate binding site, a process presumably facilitated by an electropositive amino acid net charge in proximity of the binding pocket. **b** IAA^−^-binding seemingly results in an altered configuration of the PIN transporter domain, towards the non-cytoplasmic side of the lipid bilayer. This outward-open configuration has been described for PIN8 and allows for substrate release, followed by a resetting to the inward-open configuration. **c** In the presence of excess NPA (red), IAA^−^-binding to its substrate binding site is competitively inhibited, with NPA association locking the PIN protein in its inward-open configuration.

Aiming at the topology of intrinsic membrane proteins commonly represents a major experimental challenge, and PINs were no exception. Therefore, it took the community by surprise, when in 2022 the structures of *Arabidopsis* PIN1, PIN3 and PIN8 were published in close succession by three research consortia^39–41^ all benefiting from the remarkable progress made with cryo-electron microscopy. Whilst the configuration of the disordered central loops could not be revealed, all transmembrane domain structures exhibit a high degree of similarity, reflecting a strong functional conservation between canonical (PIN1 and PIN3) and non-canonical (PIN8) PINs. All three PINs were identified as homodimers, with each monomer forming two discrete TM-helix bundles of 5. A closer examination revealed subdomains within the helical bundles; a ‘scaffold’ domain as well as a ‘transporter’ domain (Fig. 2). Key to the organization of the latter domain are conserved prolines found in helices 4 and 9, which function as ‘helix breakers’ and define a crossover point that contributes to the demarcation of the IAA-binding site. Especially, PIN structures in an ‘open-inside’ configuration share a binding cavity close to this crossover point, and adjacent to a ‘vestibule’ domain enriched for electropositive groups, potentially favouring anionic substrate attraction. In an ‘open-outside’ configuration established for PIN8, the binding site appears less buried, perhaps facilitating IAA^−^ protonation and release at the non-cytosolic membrane side. Comparison of the ‘inside-out’ and ‘outside-out’ configurations available are consistent with an elevator mode for IAA translocation (Fig. 2a, b). This mechanism, described for meanwhile numerous transporters, requires conformational changes within the transport domain structure, facilitating the transfer of IAA through lipid bilayer boundaries. Consistent with this mode of action, NPA functions as a high affinity competitive inhibitor, associating with the binding domain, thereby obstructing IAA from accessing its substrate-binding site and locking PINs in an inward facing configuration (Fig. 2c).

This ingenious body of work provided groundbreaking insights into the mechanism of PIN-dependent auxin transport. Nonetheless, quite some riddles remain; in particular regarding the active PIN transporter configuration, mechanisms of substrate recognition and energizing the transmembrane transport.

### Polarity pointing the way

A polar, subcellular localization of auxin exporters as the determinant of auxin flow directionality through tissues was already postulated by the chemiosmotic hypothesis^10,11^. PIN polarity in accordance with these predictions has been described already in some of the first PIN reports: basal PIN1 in vasculature^3^ and apical PIN2 in root epidermis cells^5^. With identification of additional PM-localized PINs, it became clear that PIN localization exhibits a considerable diversity, with different PINs adopting different polarities in a spatiotemporal context; e.g., PIN1, PIN3, PIN4, and PIN7 basal in root stele; PIN2 basal in young cortex cells but apical in the epidermis; and PIN3 apolar in the root cap columella and lateral in the endodermis. These PIN polarities correlated with, and were found to be required for the establishment of local auxin maxima or minima in many developmental processes, ranging from embryonic axis establishment to adjustments in organ growth directionality^20–24,42,43^. The ultimate demonstration that PIN polarity drives directional auxin flow came from the manipulation of PIN coding sequences, causing prominent PIN polarity switches and consequently a failure to mediate auxin flow in a given direction^44–46^.

PINs were the first polarly localized integral membrane proteins identified in plants, which made them a prominent model for polarity studies. It became clear that polarity establishment in plant cells differs substantially from animal counterparts: they are engulfed by cell walls, divide by a different mechanism and typically do not have tight junctions to delineate polar domains. Furthermore, no obvious plant homologues of known animal polarity determinants have been identified, and at least 4 distinct polar domains have been characterized at a plant cell’s PM: apical vs. basal and outer vs. inner^47^. Thus, this emerging subfield started basically from scratch with polarly localized PIN proteins representing powerful readouts for deciphering polarity mechanisms^48^.

Coincidence of PIN1 basal and PIN2 apical polarity in the very same cell, which was observed already early on, implied existence of intrinsic PIN polarity determinants^44^. Whilst PIN sequences guiding these polar decisions have not been unequivocally identified, *cis*-acting determinants have been characterized to some extent. Domain swapping, by combining portions of canonical and non-canonical PINs, demonstrated roles of the canonical PIN central loop domain for (i) PM targeting per se and (ii) polar targeting decisions^49,50^. One would envision that *trans-*acting regulators controlling PIN distribution include any component of trafficking and cargo sorting machineries. Prominent is the involvement of clathrin-mediated endocytosis (CME) in PIN polarity maintenance^51,52^. Furthermore, PINs within the PM were detected in fairly immobile clusters in the several nm-size range, which apparently contribute to polarity maintenance by limiting lateral diffusion. Pharmacological and genetic analyzes demonstrated requirements of cytoskeleton and cell wall constituents for PIN clustering, lateral mobility, and hence polarity^53–55^. There are meanwhile dozens of reports, describing defective PIN polarity arising as a consequence of genetic or pharmacological interference with cellular sorting. This reflects the large number of cellular functions that impact on PIN positioning. In contrast, meaningful and specific PIN polarity determinants are only a handful:

### GNOM

*Gnom* mutant exhibits strong embryonic polarity and patterning defects characterized by uncoordinated PIN polarities^56^. As GNOM functions as a Brefeldin A-sensitive guanine-nucleotide exchange factor for ADP ribosylation factors (ARF GEF) and is thus essential for secretory vesicle formation^57,58^ mainly at the Golgi^59^, its likely contribution is the constant PIN delivery to the centre of polar PM domains, which in combination with internalization at the edges of such domains, maintains PIN polarity^54^. There is, however, a persistent mystery with GNOM, as mutations in others, even closely related ARF GEFs, do not result in comparable polarity or developmental defects. This hints at specific, unresolved GNOM functions, possibly at the PM^60,61^.

### PINOID

**Fig. 3:**
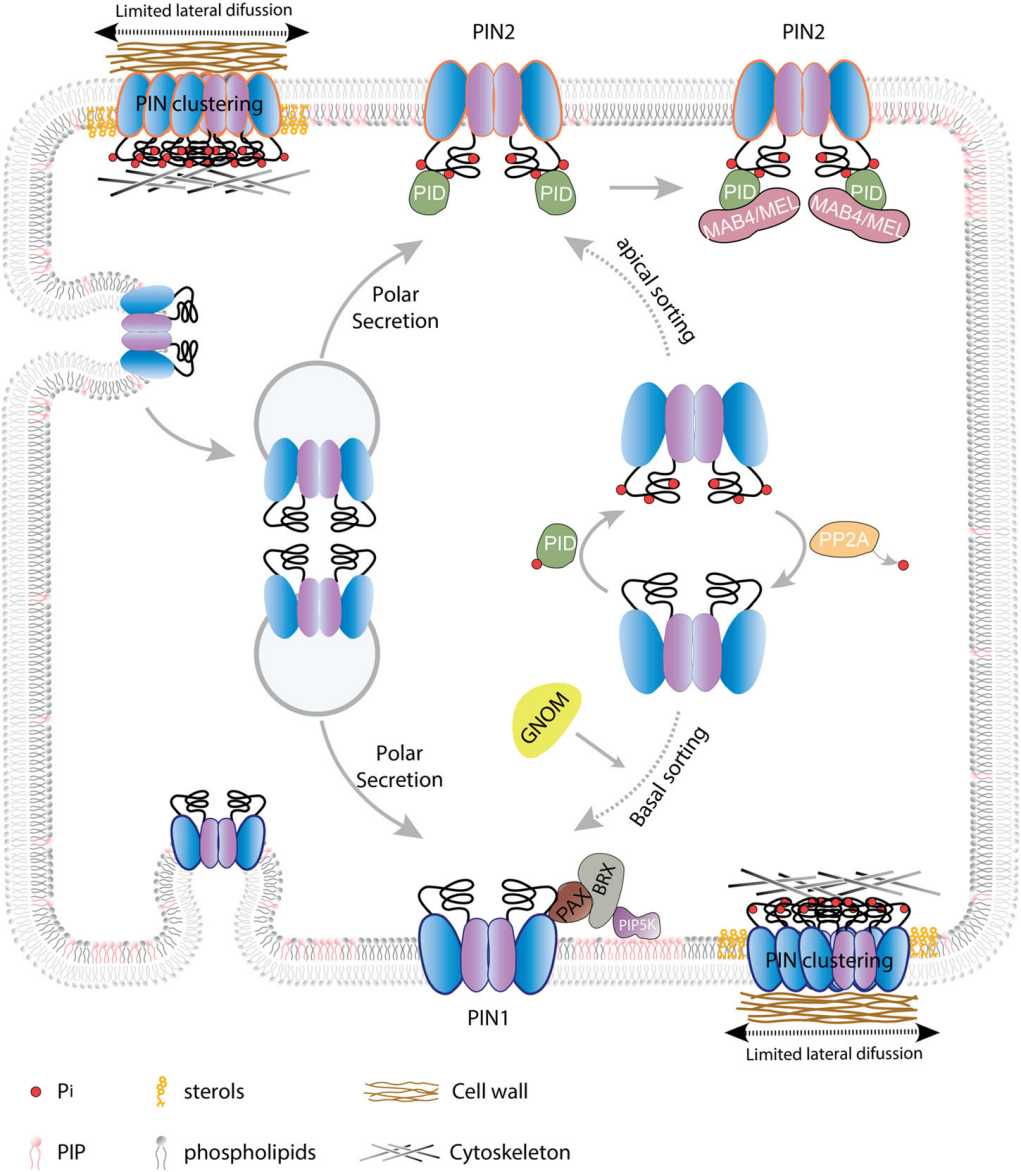
Key mechanisms of PIN polarity control. Conserved amino acid motifs in the PIN central hydrophilic loop are subject to phosphorylation (Pi, red dots) by members of the PID clade of AGCVIII-type protein kinases (green), which promotes PIN apical targeting, whereas, dephosphorylation by antagonistic PP2A protein phosphatases (amber), favors a predominantly basal sorting of PINs. It is still not entirely resolved, in which intracellular compartment such PIN phosphorylation control would take place, but it appears mechanistically linked to ARF GEF-dependent PIN recycling between sorting endomembrane compartments and polar PM domains. ARF GEF GNOM (yellow) in particular is essential for PIN recycling to basal PM domains. Once delivered to polar PM domains, PIN mobility within these domains is limited by reversible PIN clustering which has been mechanistically linked to elements of the cytoskeleton and the apoplastic cell wall. The non-clustered pool of PIN proteins is characterized by a higher mobility, reflected in its lateral diffusion within the polar PM domain. CME of PIN proteins, specifically at the outer boundaries of polar PM domains, limits further lateral PIN diffusion, thereby restricting PIN PM localization to designated domains. PIN mobility at the PM is further affected by local variations in phosphatidylinositol phosphates (PIPs) and sterol composition. This is exemplified by recruitment of PIP5K1 (purple) to the PM in dependence of PAX (brown) and BRX (grey). A strictly localized increase in PI(4,5)P_2_ levels, as a result of PIP5K1 PM recruitment is postulated to increase PIN lateral mobility and hence its endocytic sorting. On the contrary, PID-mediated PIN phosphorylation favours recruitment of MAB/MEL adaptor proteins (pink) by PINs, resulting in the formation of a PIN/MAB/MEL/PID ternary protein complex at the PM. This in turn appears to limit PIN lateral PM diffusion to maintain polar PIN localization.

*Pinoid* (*pid*) loss-of-function mutants are very similar to *pin1* mutants, developing naked knitting needle-like stems, instead of an inflorescence axis. PID encodes an AGCVIII-type protein kinase that has been linked to the control of polar auxin transport^62^. In *pid* loss-of-function alleles, PINs preferentially localize to the basal PM domains, whereas in the gain-of-function alleles to the apical sides^63^. On the other hand, loss of antagonistically acting PP2A protein phosphatase activity results in a more apical PIN localization. Importantly, PID and closely related WAVY GROWTH (WAG) kinases were found to directly phosphorylate PINs in their central hydrophilic loop^64^. Accordingly, phosphorylation-mimicking and -blocking mutations in PID-targeted phosphorylation sites within PINs lead to preferential apical and basal polarities, respectively^45,46^. All these observations collectively argue for a model, where PINs phosphorylated by PID/WAGs are delivered to the apical cell sides, whereas dephosphorylated PINs end up at the basal side (Fig. 3). Inconsistently, also PINs localized at the basal side are recognized by antibodies specific for phosphorylated PINs^28^. Additionally, another AGCVIII-type kinase, D6PK produces a PIN phosphorylation pattern, overlapping with that of PID/WAGs, however, without impacting on PIN polarity but solely on PIN auxin transport activity^65^.

PID was the first in a long line of different types of kinases, identified to phosphorylate PINs, to regulate PIN activity, stability, trafficking, and polarity. Besides AGCVIII-type kinases, also certain MAP kinases, Ca^2+^/CALMODULIN-DEPENDENT PROTEIN KINASE-RELATED KINASE and LRR-type receptor kinases were described to influence PIN subcellular targeting^66,67^. We still do not understand the interplay of all these different kinase activities, and their crosstalk with additional polarity determinants. The complexity of such regulations is exemplified by the spatial adjustments in membrane lipid biosynthesis, impacting on PIN1 polarity in protophloem cells. Here, PIN1-associated BRX and PAX AGCVIII-type kinase recruit PHOSPHATIDYLINOSITOL-4-PHOSPHATE-5-KINASE (PIP5K) to the basal PM domain^68^. As a result, spatially restricted elevated PI(4,5)P_2_ levels would establish a polar subdomain that is characterized by enhanced PIN1 endocytosis, defining PIN PM distribution, potentially uncoupled from PIN phosphorylation (Fig. 3).

### MAB4/MEL

*Macchi-bou4* (*Mab4*) mutants and mutants in homologous *MEL* (*MAB4/ENP/NPY1-LIKE*) genes show decreased apical PIN2 polarity in root epidermis cells, correlating with defects in root gravitropism. MAB4 and MELs exhibit a polar localization largely overlapping with PINs in a range of tissues/cell files^69^. Notably, PINs, PID, and MAB4/MEL directly interact, with PID-mediated PIN phosphorylation increasing MAB4/MEL recruitment, which in turn would recruit PID, thus forming a self-reinforcing mechanism, to limit PIN lateral diffusion and maintain its polar PM distribution^70^ (Fig. 3). Spatially separated requirements of PID/WAG-dependent PIN phosphorylation within the cell for both, apical sorting and maintenance within the polar domains, together with control of PIN transport activity by both PID and D6PK would reconcile the established role of PID/WAGs in PIN targeting with the presence of phosphorylated PINs at apical as well as basal PM domains.

### WAV3/WAVH

A combined loss of *WAVY ROOT GROWTH 3* (*WAV3*) and of *WAV3 HOMOLOG 1* and *2* (*WAVH1/WAVH2*) function leads to a less auxin-responsive and highly agravitropic root growth^71^. These phenotypes can be well explained by the distinctive polarity shift of apically localized PIN2, which in the mutant gets routed to the basal PM domain, abolishing shootward auxin transport in the root tip^72^. Strikingly, no such aberrations were detected for basally localized PIN1, indicative of *WAV3/WAVH* acting only on a subset of PINs. Insights into mechanisms by which WAV3/WAVH RING-finger type ubiquitin E3 ligases control PIN polarity are complicated by the identification of another function for *WAV3/WAVH* and for the rice *WAV3* homolog *SOIL-SURFACE ROOTING 1* (*SOR1*), acting in ubiquitination and turnover of non-canonical Aux/IAA proteins^73,74^. This linked WAV3/WAVH/SOR1 function to transcriptional auxin signalling downstream of *TRANSPORT INHIBITOR RESPONSE 1/AUXIN-SIGNALING F-BOX* (*TIR1/AFB*) pathways^73^ and to TRANSMEMBRANE KINASE 1 (TMK1)-mediated auxin signalling^74^. Mechanisms underlying such diversified roles of WAV3/WAVH/SOR1 are entirely unknown.

The last 25 years saw an emergence of the plant cell polarity field largely depending on PINs as prominent model. Nonetheless, classical genetic approaches seem to reach their limits, likely because of essential roles or functional redundancy of polarity determinants. Presumably, more advanced genetic and biochemical approaches are needed to make further breakthrough in this field.

### Trafficking for polarity and beyond

Canonical PINs, as intrinsic PM proteins, undergo tightly controlled intracellular trafficking. This led to the very important insight that actually most integral PM proteins in plants undergo constitutive endocytic recycling^75^. In case of PINs this not only ensures their proper delivery and maintenance at designated polar domains^54^, but also allows dynamic polarity changes to rapidly divert auxin fluxes within tissues^23,54,76^.

As any protein destined for the PM, canonical PINs undergo a succession of sorting steps, guiding their controlled passage from the ER via Golgi and post-Golgi sorting vesicles. PINs were also among the first demonstrated cargoes for CME^77^. Once internalized, PINs have a number of choices, namely (i) recycling to the original polar domain at the PM^54^; (ii) delivery to another PM domain via transcytosis^78^ and (iii) rerouting into late endosomes for their vacuolar targeting and proteolytic degradation^79^.

High-resolution imaging revealed highly polarized exocytotic PIN2 delivery to the centre of the designated polar domain. Strikingly, subsequent CME-mediated internalization occurs mainly at the lateral limits of these domains^54^ (Fig. 3). Such spatially confined PIN internalization and polarized recycling along with limited PIN lateral diffusion within the PM would ensure polar maintenance in the absence of tight junctions, not only for PINs but additional polar cargoes as well^47^. Accordingly, perturbation of PIN endocytosis causes severe PIN polarity defects^51,80^. Forward^81^ and especially reverse genetics approaches led to the identification of elements of the PIN trafficking machinery. For exocytic routes, these encompass components of the vesicle-tethering exocyst complex^82,83^, Rho of plants (ROP) GTPases such as BFA-VISUALIZED EXOCYTIC TRAFFICKING DEFECTIVE (BEX)^84,85^ and the retromer for the endocytic route towards the vacuole^86,87^. Further factors relate to CME, as both, structural elements and factors guiding clathrin-coated vesicle formation were linked to the regulation of PIN internalization^51,88^.

Characterizing PIN trafficking was greatly aided by pharmacological approaches. Specifically, the inhibitory effects of the fungal toxin Brefeldin A (BFA) on a subset of ARF GEFs that function in vesicle formation, along with engineering ARF GEF sensitivity to BFA were extensively exploited^57^. BFA-induced PIN accumulation in Golgi-derived ectopic vesicular structures, versus their release from such ‘BFA compartments’ upon drug washout^75^, provided insights into protein trafficking in general, and PIN trafficking in particular. Whilst GNOM and GNOM-LIKE1 ARF GEFs have been associated with early steps of polar PIN1 trafficking already along the ER-Golgi passageway^89^, the most relevant roles of GNOM appear to be at the Golgi^59^ in PIN polar recycling to the basal domain^78^ and still elusive functions at the PM^60,61^ (Fig. 3). Additional ARF GEFs, namely BIG1 (BREFELDIN A-INHIBITED GUANINE NUCLEOTIDE-EXCHANGE PROTEIN 1), BIG2/BEN3 (BFA-VISUALIZED ENDOCYTIC TRAFFICKING DEFECTIVE 3), BIG3, BIG4 and BIG5/BEN1/MIN7 (HOPM1 INTERACTOR 7) have been assigned various roles in guiding endosomal trafficking and vesicular sorting of PINs along the trans-Golgi network/early endosomal route^81,90–92^. Notably, whilst the function of ARF GEFs as well as of VASCULAR NETWORK DEFECTIVE 3/SCARFACE (VAN3/SFC), an ARF GTPase Activating Protein (ARF GAP) could be clearly associated with different PIN trafficking steps^93^, the identity of the matching small ARF GTPases remains unknown, hinting at substantial functional redundancies within this large protein family^94^.

How exactly PIN trafficking is mechanistically linked to polar sorting decisions remains obscure. For example, it was proposed that PID-mediated PIN phosphorylation guides PIN recruitment into distinct apical vs. basal ARF GTPase-controlled sorting pathways. This is underlined by consequences of long-term ARF GEF inhibition leading to the apicalization of basal PINs, whereas there is only limited BFA responsiveness of apical PINs^95^. While the details of this mechanism remain unclear, it establishes causal links between PID and GNOM in control of PIN polarity.

Selective trafficking also controls PIN degradation, a process prominently contributing to regulation of directional auxin fluxes in different contexts^79,96,97^. Reversible K63-linked polyubiquitination of PINs induces vacuolar targeting followed by proteolytic degradation^79^. Ubiquitinated PIN2 recognition signals sorting via Late Endosomes (LE)/Multivesicular Body, mediated by the ENDOSOMAL SORTING COMPLEXES REQUIRED FOR TRANSPORT machinery and modulated by elements of the retromer complex and CYTOPLASMIC LINKER ASSOCIATED PROTEIN (CLASP)^86,87,98–100^. Whilst mechanistic implications of PIN ubiquitination have been characterized, ubiquitin E3 ligases involved have not been identified to date, obstructing conclusions on the biological role of such PIN modification. Next to ubiquitination, vesicle identity has been demonstrated to impact on the fate of PINs bound for degradation. FORMATION OF APLOID AND BINUCLEATE CELL 1C, a PIP3P5-kinase physically interacts preferably with hypo-phosphorylated PINs. As a result, membrane-intrinsic PtdIns(3)P converted to PtdIns(3,5)P_2_, promotes the transition of Early Endosome to LE identity, thereby affecting PIN vacuolar targeting^101^. Links between PINs and membrane environments thus surfaces as a reoccurring theme^58,68^, which definitely deserves to be characterized in further detail.

### Endogenous signals converging

With local auxin maxima and gradients established as versatile mechanism underlying many growth and developmental processes^102^, it is no surprise that multiple endogenous and environmental signals converge on dynamic PIN-dependent auxin distribution, by targeting PIN expression, subcellular trafficking, and polarity^103^.

Auxin itself represents a prominent signal, affecting PIN polarity and trafficking, thereby connecting spatiotemporal variations in auxin homeostasis to adjustments in auxin transport. On the one hand, auxin, via canonical TIR1/AFB signalling, triggers PIN2 ubiquitination as a signal for its sorting towards the lytic vacuole and proteolytic turnover^96,104^. On the other hand, auxins, in particular synthetic ones such as 1-Naphthaleneacetic acid (NAA) interfere with internalization of PM cargos, including PINs^105^ by a non-transcriptional mechanism that may involve the AUXIN BINDING PROTEIN 1 (ABP1)/TMK signalling pathway^106^. Originally proposed as targeting CME, more advanced microscopy techniques did not reveal any NAA effect on individual CME events, but rather on the endomembrane system and endocytic trafficking in general^107,108^, presumably involving phosphorylation of Myosin XI motor protein, downstream of ABP1/TMK auxin perception^109,110^.

Feedback regulation, by which auxin promotes capacity and directionality of its own transport has been proposed as a necessary prerequisite of the so-called auxin canalization, a process underlying the ability of plants to flexibly initiate or regenerate vasculature^111^. Auxin canalization proposes auxin-transporting channels polarized away from the auxin source, established in an initially homogeneous tissue to guide vasculature formation^112^. Indeed, a gradual formation of channels marked by expression of polarized PINs is observed in different contexts such as: (i) connecting leaf or flower primordia at the shoot apical meristem^42^ as well as lateral buds released from dormancy^113^ with the pre-existing vasculature; (ii) during vasculature regeneration following wounding^114,115^; and in (iii) leaf venation^116,117^. Recently, molecular determinants transmitting the auxin signal in canalization and PIN polarization have been characterized to require both, nuclear, canonical TIR1/AFB and apoplastic ABP1/TMK auxin perception mechanisms^110,118^. Downstream, the WRKY23 transcription factor^119^ and CANALIZATION-RELATED AUXIN-REGULATED MALECTIN-TYPE RECEPTOR-LIKE KINASE, together with CANALIZATION-RELATED RECEPTOR-LIKE KINASE were found instrumental for PIN phosphorylation, guiding their polarity during canalization^119^. PINs and auxin feedback on them, during canalization, are presumably targeted by strigolactone hormones^120,121^ to regulate shoot branching as well as formation and regeneration of vascular tissue^113,114^.

Cytokinins (CK) represent a class of hormones intimately linked to auxin, as they function as antagonists in root/shoot differentiation^122^, root gravitropism^123,124^ and lateral root formation^125^. Parts of this regulation may occur via CK promoting PIN vacuolar degradation and establishment of basal cellular polarity in cells of emerging lateral root primordia^125^. On the other hand, Gibberellic acid (GA) appears to have an opposite effect; inhibiting PIN delivery to the vacuole via the canonical DELLA pathway^99^. This mechanism may contribute to root gravitropism, in which GA accumulates at the lower root side^126^, where it may stabilize PIN2 and auxin flow along the lower side of the root. Also brassinolide signalling appears to converge on regulation of PIN2 during root gravitropism, antagonizing its differential endocytic sorting and vacuolar degradation, crucial for resetting differential auxin transport upon completion of gravity-induced redirection of root growth^127^. Salicylic (SA) and Jasmonic (JA) acids are another pair of hormones, exhibiting antagonistic roles in signalling events associated with biotic and abiotic stresses. SA interferes with Brefeldin A-sensitive endocytic trafficking of PINs^128^. This occurs via SA binding to PP2A, inhibiting its activity and enhancing PIN2 phosphorylation^129^. Along these lines, anti-inflammatory painkillers structurally related to SA, such as aspirin, were found to target TWISTED DWARF1(TWD1)-regulated actin dynamics and auxin transport^130^. Exogenous JA application also impacts on PIN trafficking in a dosage-dependent manner and in conjunction with auxin signalling^131^, but the biological significance of this effect is less characterized. PP2A phosphatase activity is also instrumental for transmission of abscisic acid (ABA) signals on PIN trafficking. Here, the canonical PYRABACTIN RESISTANCE (PYR)/PYR-LIKE receptors interact with PP2A, presumably affecting PIN phosphorylation and thus localization or trafficking^132^. Finally, secretory signalling peptides, belonging to the GOLVEN family were found to stabilize selected PINs, potentially by antagonizing their endocytic sorting from the PM^133^.

Collectively, most characterized signalling trajectories have some defined impact on PIN-mediated auxin transport. This of course reflects a certain bias in the PIN-centred studies, but it certainly highlights the central role of PINs in many developmental processes, along with multiple possible upstream entry points in processes of endocytosis, trafficking, and cell polarity.

### Environmental cues converging

Constitutive endocytic PIN recycling and PIN polarity are also targeted by environmental cues, especially where rapid adjustments of growth and development are integral elements of environmental adaptation^134^.

One of the fastest adaptation responses happens to be root gravitropism, with the directionality of root growth adjusted within a few minutes^135,136^. Gravity perception in higher plants occurs by sedimentation of starch-containing amyloplasts (statoliths) in gravistimulated columella root cap cells, followed by PIN polarization at the cells’ bottom sides via ARF GEF-mediated transcytosis^23,137^. This asymmetry in PIN localization would redirect auxin fluxes towards the lower root side, with elevated auxin levels inhibiting cell elongation in comparison to the upper side, causing root downward bending. Statolith sedimentation is translated into PIN relocation by action of NEGATIVE GRAVITROPIC RESPONSE OF ROOT (NGR)/LAZY1-LIKE proteins, which localize to the statoliths and the PM and also polarize to the bottom of columella cells, following gravistimulation^138–141^. NGR relocation and auxin accumulation at the lower root site occur very rapidly, even before any visible polarization of PIN proteins. This relates to a rapid, gravity-induced relocation of D6PK kinase, which could phosphorylate and activate PINs, immediately redirecting auxin flow regardless of PIN polarization^141^. This is in line with the importance of PIN phosphorylation for root gravitropism, however, no strong gravitropic defects have been reported for *d6pk* mutants^142^.

The initial auxin asymmetry in the root tip needs to be propagated towards the root meristem elongation zone, where differential cell elongation defines root bending. This auxin flow from the tip is mediated mainly by PIN2. Following gravistimulation, PIN2 expression develops asymmetry with higher levels at the lower vs. the upper root side. This likely results from differential vacuolar targeting, a process that coincides with variations in the overall ubiquitination and degradation of PIN2^96,137,143^. Resulting transient adjustments in PIN2 abundance likely reinforce the initial auxin asymmetry with more auxin flow along the lower root side and might as well participate on resetting of organ bending. Related mechanisms operate during gravity responses in lateral roots. Here, the gravitropic set point angle (GSA, describing maintenance of non-vertical directional lateral root growth with respect to the direction of the gravity vector) involves transient lateral expression gradients and cellular polarization of PIN3 and PIN7, linked to the PIN phosphorylation status by subtle variations in the activity of PP2A protein phosphatase^144,145^.

Negative shoot gravitropism also operates via starch granule sedimentation, followed by PIN3 relocation to the bottom PM domains of endodermal cells, which leads to auxin accumulation at the shoot’s lower side and ultimately to upward shoot bending^76^. This auxin accumulation triggers a further PIN3 relocation reminiscent of auxin-induced PIN polarization during canalization, which re-establishes PIN3 symmetry and hence balances auxin distribution upon termination of shoot bending^146^.

Phototropism involves blue light perception by PHOT1 and PHOT2 phototropins, eventually leading to PIN3 polarization in hypocotyl endodermis cells at the PM sides opposite to the incoming light stimulus. Together with adjustments in PIN phosphorylation, this provides a possible mechanism for generating an auxin asymmetry during phototropic bending^147^. Nonetheless, the timing, causality, and underlying mechanism are even less elucidated than in case of gravitropism^148^.

Another major adaptive response to light quality, termed shade avoidance, describes plants’ responses when competing for their place in the sun. Such accelerated upward growth of shoot-derived organs may involve a combination of adjustments in auxin biosynthesis and PIN3 lateralization in endodermis cells, promoting auxin flow towards the shoot epidermis and consequently shoot elongation^149^.

Dynamic polar PIN positioning as a recurring theme in directional growth responses has also been detected during apical hook formation/opening in etiolated seedlings. Here, PIN3- and PIN4-mediated lateral transport to the concave, inner hook side, drives auxin accumulation. In addition, elevated PIN expression at the convex, outer side of the hook has been proposed to drain auxin from this area acting as determinants of a transient auxin minimum^150^. Light-induced opening of the apical hook in turn requires a resolution of asymmetries in PIN expression and polarity, a process that seemingly involves PIN phosphorylation control^151^, CME^152^ and elements of the cytoskeleton^153^.

Apart from their numerous roles in the implementation of environmental stimuli into pre-determined developmental programs, PINs do also impact on plant adaptation as an integral element of responses to stressful environments. This involves nastic leaf movements, to facilitate leaf cooling in warm environments, by means of PID-dependent control of PIN3 polarity in leaf petioles^154^. Avoidance of saline soil environments, in contrast, is mediated by enhanced asymmetric CME of PIN2 in those portions of the root meristem proximal to elevated saline levels. This might be triggered by salt-induced activation of phospholipase D, with elevated PA levels favouring establishment of PIN2 and hence auxin gradients to facilitate directional root growth away from areas of higher salinity^155^. Such salt-responsive mechanisms may be related to more general effects of osmotic or oxidative stresses on the interplay between endo- and exocytosis, impacting PIN levels at the PM^156,157^.

This extensive yet incomplete list of examples, clearly shows that adjusting distinct aspects of PIN-dependent auxin transport is a common mode of action, by which environmental cues and conditions are translated into modulation of plant development and physiology.

### Evolution

**Fig. 4:**
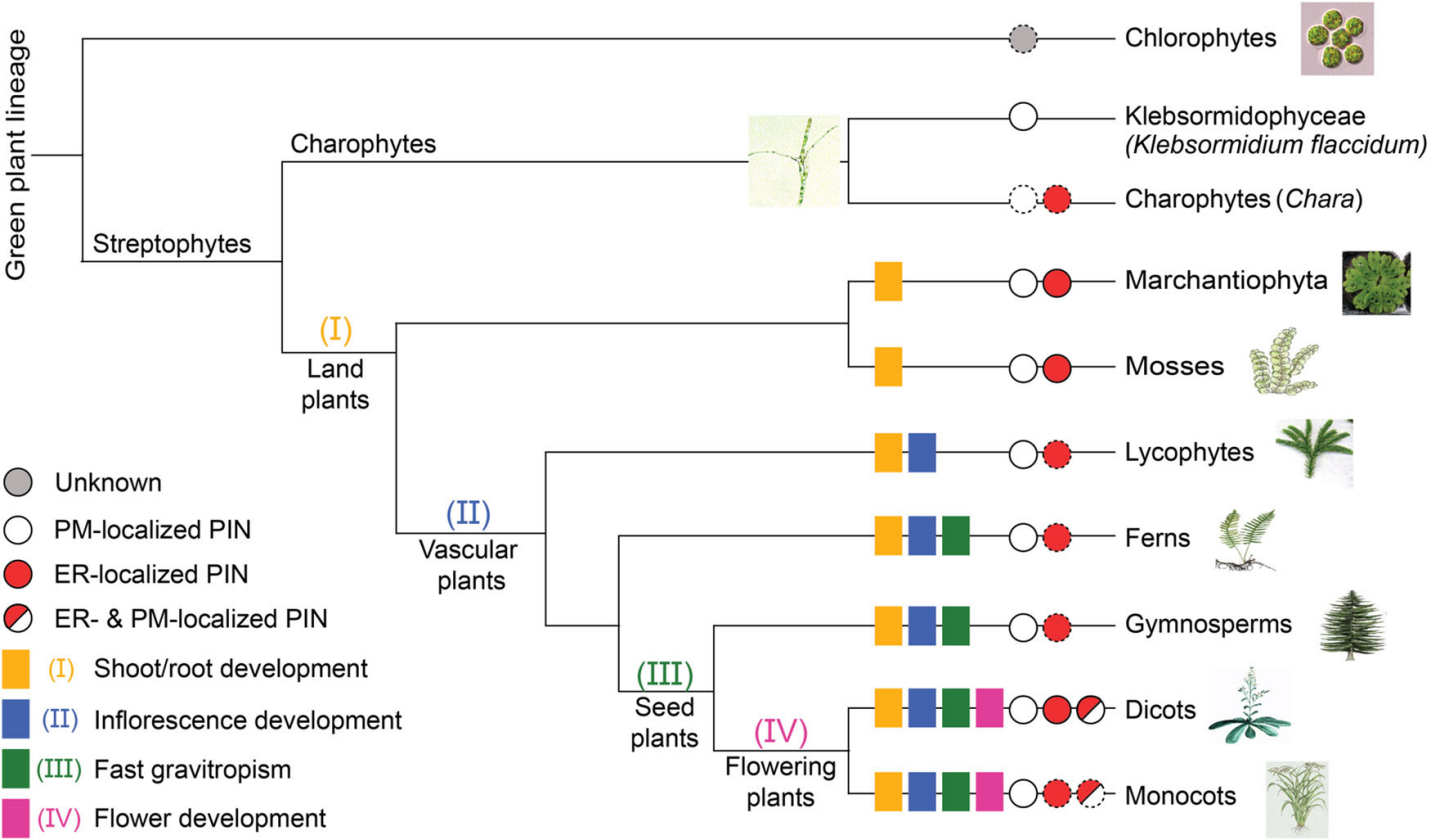
Phylogeny of the PIN family in the green plant lineage. PINs functioning as auxin exporters have been demonstrated for Streptophytes; in Charophyte algae based on the characterization of the PM-localized, auxin-exporting *Klebsormidium* PIN, and in land plants, where already in bryophytes, both PM- and ER-localized PINs are present. In the latter, PM PINs underwent several functional innovations, endowing PINs with the ability to mediate (I) aerial/underground tissue development, (II) inflorescence formation, (III) fast root gravitropism linked with apical PIN localization, and (IV) flower formation; as shown when introduced into the model Arabidopsis. In Arabidopsis PIN6 endogenously present simultaneously at the PM and ER was characterized. Based on in silico sequence analyses, but yet lacking experimental confirmation (indicated by dotted circles), we assume the presence of (i) PM/ER-localized PINs also in monocots; (ii) ER-localized PINs along the whole land plant lineage; (iii) scattered presence of PIN-like transporters in chlorophytes; and (iv) independent radiation of PINs in Chara. A subset of images used for this Figure has been published previously by Zhang and colleagues^163^, licensed under a Creative Commons Attribution 4.0 International License (https://creativecommons.org/licenses/by/4.0/).

Directional, intercellular PIN-dependent auxin transport and resulting asymmetric auxin distribution as versatile developmental mechanism seem unique to land plants. When this process first emerged in the plant lineage and how it was co-opted to underlie ultimately most of higher plants’ development remains unclear. This mystery is linked to the enigma of the origins of auxin itself. Was it originally a toxic by-product of metabolic processes? A poison used to weed the competition in the surroundings? A signal originally used by microbes to interact with plants? We do not have many clues so far^158^. We know that already long before the rise of complex morphologies, at the onset of the streptophyte lineage that gave rise to land plants, PIN proteins were present and capable of exporting auxin^159^, as shown for the single PIN present in the simple, filamentous alga *Klebsormidium* (Fig. 4). This ‘prototypal’ algal PIN shows remarkably specific auxin transport activities as well as BFA-sensitive subcellular trafficking, but does not adopt any polar PM distribution^160^. Unfortunately, the actual physiological role of PINs in algae such as *Klebsormidium* is not understood, largely due to inaccessibility of this model to genetic engineering. It is, however, clear that morphologically more complex algae, such as *Chara* encode for multiple PIN proteins, likely reflecting an independent radiation^161^. In early diverging land plants, such as the bryophytes *Marchantia* or *Physcomitrella*, PINs already radiated into PM- versus ER-localized PINs, and acquired a distinctively defined subcellular polarity, linked to individual developmental and adaptive processes^162^ (Fig. 4).

In the land plants, PINs underwent functional innovations, which occurred at three distinct evolutionary steps, the origin of (i) land plants, (ii) vascular plants, and (iii) flowering plants. They endowed different PINs with cell type- and tissue-specific expression and different subcellular, polar localizations^50^ (Fig. 4). A fascinating example is the acquisition of a specifically apical PIN localization in root epidermis cells at the onset of seed plants; a necessary prerequisite for rapid root gravitropic growth responses^163^. Also, the numerous regulations of trafficking, stability and polarity by endogenous and environmental cues must have been acquired in course of seed plant evolution, but how, when and in which order, remains unclear. Nonetheless, it was these regulations, gradually evolving, that made PIN-mediated auxin transport the unique, versatile system regulating so many aspects of plant development.

### Concluding remarks

Some 25 years after their initial discovery it is now evident that PIN transporters act as central players of the plant-specific mechanism regulating development, throughout the entire life cycle. This PIN-dependent auxin distribution network also integrates various external cues, thus adapting plants’ growth and development to their environment. This knowledge, together with the currently ongoing in-depth characterization of PINs, holds great promises for precision breeding approaches, aiming at fortifying crop performance even under unfavourable environmental conditions. Somewhat unexpectedly, PIN research might not be limited to the plant kingdom. The recent characterization of the LYCHOS transport domain as a human PIN ortholog, points towards common substrate or transport principles in the different kingdoms, actually originating from PINs^164^. Further elucidation of commonalities and differences between these orthologs, will definitely produce essential insights into the role of PINs as master regulators of plants, and beyond.
